# Vulnerability to Food Insecurity in Urban Slums: Experiences from Nairobi, Kenya

**DOI:** 10.1007/s11524-014-9894-3

**Published:** 2014-08-30

**Authors:** E. W. Kimani-Murage, L. Schofield, F. Wekesah, S. Mohamed, B. Mberu, R. Ettarh, T. Egondi, C. Kyobutungi, A. Ezeh

**Affiliations:** 1 African Population and Health Research Centre, Nairobi, Kenya, P.O. Box 10787, 00100 Nairobi, Kenya; 2 Save the Children, UK; formerly of Concern Worldwide, Kenya, Dublin, Ireland

**Keywords:** Urban poor, Sub-Saharan Africa, Food security, Kenya, Crisis, Vulnerability

## Abstract

Food and nutrition security is critical for economic development due to the role of nutrition in healthy growth and human capital development. Slum residents, already grossly affected by chronic poverty, are highly vulnerable to different forms of shocks, including those arising from political instability. This study describes the food security situation among slum residents in Nairobi, with specific focus on vulnerability associated with the 2007/2008 postelection crisis in Kenya. The study from which the data is drawn was nested within the Nairobi Urban Health and Demographic Surveillance System (NUHDSS), which follows about 70,000 individuals from close to 30,000 households in two slums in Nairobi, Kenya. The study triangulates data from qualitative and quantitative sources. It uses qualitative data from 10 focus group discussions with community members and 12 key-informant interviews with community opinion leaders conducted in November 2010, and quantitative data involving about 3,000 households randomly sampled from the NUHDSS database in three rounds of data collection between March 2011 and January 2012. Food security was defined using the Household Food Insecurity Access Scale (HFIAS) criteria. The study found high prevalence of food insecurity; 85 % of the households were food insecure, with 50 % being severely food insecure. Factors associated with food security include level of income, source of livelihood, household size, dependence ratio; illness, perceived insecurity and slum of residence. The qualitative narratives highlighted household vulnerability to food insecurity as commonplace but critical during times of crisis. Respondents indicated that residents in the slums generally eat for bare survival, with little concern for quality. The narratives described heightened vulnerability during the 2007/2008 postelection violence in Kenya in the perception of slum residents. Prices of staple foods like maize flour doubled and simultaneously household purchasing power was eroded due to worsened unemployment situation. The use of negative coping strategies to address food insecurity such as reducing the number of meals, reducing food variety and quality, scavenging, and eating street foods was prevalent. In conclusion, this study describes the deeply intertwined nature of chronic poverty and acute crisis, and the subsequent high levels of food insecurity in urban slum settings. Households are extremely vulnerable to food insecurity; the situation worsening during periods of crisis in the perception of slum residents, engendering frequent use of negative coping strategies. Effective response to addressing vulnerability to household food insecurity among the urban poor should focus on both the underlying vulnerabilities of households due to chronic poverty and added impacts of acute crises.

## Background

Urban poverty is increasingly becoming important in sub-Saharan African cities, as a result of rapid urbanization amid poor governance, lack of political will and poor planning. While sub-Saharan Africa has had sustained economic growth in the last one to two decades, the region still has the highest rate of poverty with close to half of the population living on only US$1.25 a day. The region accounts for 30 % of the most poor in the world.^1^ Rapid growth of the urban population, poor governance and lack of political will and weak economic growth since the 1980s with Structural Adjustment Programs have impeded governments’ ability to provide basic services and decent living conditions in urban areas in SSA. As a result, the majority (estimated at 62 % in 2010) of urban residents in this region live in overcrowded slums and shantytowns,^2^ characterized by poor livelihood opportunities, inadequate water and sanitation infrastructure, very poor housing conditions, and limited education, health and other social services.^3^^–^ 6

Urban centers are characterized by cash-based economies, with even the extreme poor accessing most of their basic needs through the market. Access to an income is therefore essential for household well-being and food and nutrition security of the urban poor is highly dependent on their livelihoods. Recent analysis of household expenditures in urban poor settings has shown that the urban poor living on less than US$1 per day spend more than half of their budget on food.^7^^,^ 8 Most of the urban poor rely on wage labor with, for example in Kenya, men mostly employed in low-wage casual or temporary jobs in industries or construction sites and women mostly employed as domestic workers. They rely on very low levels of income, yet they sometimes pay more for goods and services compared to their non-slum counterparts, leading to high level of vulnerability.^9^^–^ 11

Evidence indicates that the concentration of poverty and malnutrition is now shifting from rural areas to urban areas. Using data collected in the late 1970s to 1990s in eight countries (for poverty) and 14 countries (for malnutrition) in the developing world, Haddad et al. show that the absolute number of poor and undernourished individuals living in urban areas increased in a majority of the countries.^12^ Some of the factors that contribute to food insecurity in urban areas include (i) an overdependence on purchased food commodities, (ii) a lack of sufficient livelihoods, (iii) rapid reductions in peripheral agricultural land and (iv) adverse impacts of climate change.^13^ What is different between the urban and the rural poor is the dominance of the cash economy over access to food in the former, which links urban food systems to poverty and vulnerability to food insecurity.^14^ The situation is made worse by the lack of formal safety nets in urban informal settlements, coupled with the lack of political will and commitment to coping with food insecurity and other problems, leaving the burden to individuals and households.^15^

Worsening food insecurity is a key characteristic of most crises and a collapse in a population’s food security leads to increased morbidity and mortality. The nature, extent, and duration of coping strategies employed by the urban poor in the face of food crisis and consequent food insecurity, will determine the magnitude and severity of their suffering.^16^ Understanding vulnerability to food insecurity in urban contexts is therefore central to understanding and identifying crises in this population. Garret and Cohen present a thorough examination of the processes leading to food insecurity among the urban poor using the food price crisis in 2008 as lenses.^11^^,^ 17 As net buyers of food, the urban poor are strongly affected by even marginal food price increases; they also have less flexibility than their rural counterparts to shift to other staple foods when prices rise.

Using the UNICEF conceptual framework on causes of malnutrition as a guide, Ruel et al. described the key issues surrounding food and nutrition security in urban areas. They argue that urban food markets can be relatively inefficient, as the markets are ill-equipped and not well located to handle the volumes of food that pass through them. Urban retail outlets are generally small and scattered, which allows easy access by the urban poor but makes minimal gains from economies of scale. Urban poor consumers are often unable to purchase food in larger quantities and can therefore end up paying higher prices per unit as they purchase foodstuffs on a daily basis. This purchasing pattern also leads to heavy reliance on street foods, both because of the income patterns and time constraints which make cooking at home difficult for many urban poor households. Evidence from some countries suggests that the poorest households spend proportionally more on street foods, while the consumption of street foods has negative nutritional and food safety implications.^18^

Given the vulnerability of the urban poor to crises, this study describes the experiences of urban poor residents in the informal settlements of Nairobi with food insecurity, with a particular focus on vulnerability during security crisis, particularly the case of 2007/2008 postelection crisis in Kenya. The value of this study is in documenting slum residents’ own experiences and perceptions of food insecurity and how they cope with this defining feature of their lives. This would help in understanding how both chronic and acute food security can be detected and addressed in urban poor communities, for timely and effective humanitarian response.

## Data and Methods

### Study Setting

The study was undertaken in two urban slums in Nairobi, Kenya: Korogocho and Viwandani. The two slums are located about 10 km from the city center, and about 7 km from each other. The two slum areas, are densely populated (63,318 and 52,583 inhabitants per square km, respectively). They are characterized by lack of basic infrastructure, high unemployment rates, poor water and environmental sanitation, poor housing, insecurity, violence, and poor health indicators.^3^^,^ 4 ^,^ 19 Socioeconomic status of the two slums differs slightly. Located in the industrial area, Viwandani residents have relatively higher levels of education and employment as the slum attracts migrant workers to the surrounding industries. Additionally, it has a higher prevalence of single-person households. Korogocho on the other hand has a more stable population, with residents having generally lived there for several decades, the slum having been in existence for over four decades. Korogocho also has greater co-residence of spouses, and the family size is generally bigger (average of 3.0 people per household compared to 2.3 in Viwandani).^20^ The study was nested within the Nairobi Urban Health and Demographic Surveillance System (NUHDSS), run by the African population and Health Research Center (APHRC). The NUHDSS follows about 70,000 individuals in close to 30,000 households in these two communities. More details about the study setting are available elsewhere.^20^

### Data

This paper draws data from a wider on-going study aimed at developing indicators for surveillance of urban emergencies being conducted in urban areas in Kenya.^21^ The study uses a mixed method approach, triangulating both qualitative and quantitative data sets. The quantitative data was collected through a household interviewer-administered survey conducted in three rounds in March/April 2011, July/August 2011 and December 2011/January 2012, involving 1045, 1118 and 1047 households, respectively. The study households were randomly sampled from the NUHDSS database in each round. The three rounds of data collection happened during a period of rising commodity prices in Kenya. In all selected households, the head of the household (or his/her representative) was first approached to consent to the household participating in the household interview and sign a prewritten consent form. A credible respondent from the household, mostly the household head or the spouse was then interviewed. Questions were asked in various domains including water and sanitation, food security, livelihoods, health status, interpersonal relationships, personal and property security and housing and tenure. In this paper, we focus on questions in the food security domain, and to some extent personal security and livelihoods domains. A set of questions related to food security including main source of food for the household, consumption of street foods, food intake including number of meals, types of food consumed by household members and food access were asked.

The qualitative study, conducted in November 2010 focused on how the urban poor defined a normal situation in terms of the various domains mentioned above; how they defined, perceived and experienced crisis, focusing specifically on a recent crisis; and coping mechanisms during a normal and a crisis situation. The qualitative study involved 10 focus group discussions (FGDs) with community members and 12 key informant interviews (KIIs) with community leaders. Participants were selected primarily using purposive sampling methods. Each of the FGDs consisted of 8–10 people. The groups comprised younger men (15–24), older men (25+ years), female household heads, married women and unmarried young women (15+ years). The Key Informant Interviews (KIIs) were conducted among the community leaders including teachers, religious leaders, community-based organization (CBO) leaders, women groups, youth group representatives, village leaders, administrative leaders and health professionals. Interviews were recorded using digital recorders and the interviewers also took notes during the interviews.

### Data Analysis

Data from the three rounds of data collection were pooled for the analysis. Close to 10 % of the households were involved in at least two of the three rounds of data collection as the sampling for each round was random. Food security was defined using a modified Household Food Insecurity Access Scale (HFIAS).^22^ A set of questions (Appendix) that relate to three different domains of food insecurity (access) were used in the score. Eight as opposed to the usual nine questions were used. One of the questions, question 4 on the usual list, “Did you or any household member eat food that you preferred not to eat because of lack of resources to obtain other types of food?”, was dropped during the pilot phase as it was not considered different in meaning from another question on the list by the study respondents; question 2, “were you or any household member not able to eat the kinds of foods you preferred because of a lack of resources”. The domains include: (i) *Anxiety and uncertainty about the household food supply* with regards to whether one worried that the household would not have enough to eat; (ii) *insufficient quality* in terms of variety and preferences of the type of food the household accessed; and (iii) *insufficient food intake* in terms of reducing quantity of food eaten in a meal and number of meals. The HFIAS score was used to categorize food security into four categories including food secure, mildly food insecure, moderately food insecure and severely food insecure in order to determine prevalence of varying levels of food insecurity. For the purposes of analysis in this paper, mildly and moderately food insecure categories were combined into one category.

Descriptive quantitative data analysis was done to determine frequencies and means. Statistical analysis was done using Stata version 11.1 (StataCorp LP, College Station, TX, USA). Further bivariate and multivariate analysis of factors associated with food security was conducted by slum of residence. Logistic regression was used with food security (whether food secure or insecure) as the dependent variable. The category “food insecure” was a composite of three categories “mildly”, “moderately” and “severely” food insecure. We modelled the odds of being “food secure” as the outcome in the analysis. For explanatory variables, we included variables likely to influence food security status that were available which included level of income (self-reported); type of livelihood source; size of household (determined using number of people living in the household); dependence ratio (ratio of members not working vs. those working); proportion of households with a member ill in the last 2 weeks of the interview; whether the household had experienced any type of shock (fire, floods, mugging/stabbing, burglary, eviction, property destruction, rape/sodomy) within the last 4 weeks of the interview; perception of insecurity status within the neighborhood (within the last 4 weeks of the interview); and slum of residence. Household size and dependence ratio were used as continuous variables in the analysis.

For the qualitative data, all interviews were translated to English and transcribed to MS Word files. The transcripts were then coded in MAX QDA and the analysis was done using a constant comparison method.^23^^,^ 24

## Results

### Prevalence of Food Insecurity in the Slums

The analysis indicates high prevalence of food insecurity. The prevalence of severe food insecurity for the pooled analysis of the three rounds was 50 %, mild-moderate food insecurity was 35 % while only 15 % of the households were food secure based on the Household Food Insecurity Access Scale (HFIAS). The situation of food insecurity was worse in Korogocho slum compared to Viwandani, with 64 % of households being severely food insecure compared to 33 % in Viwandani, and only 8 % being food secure compared to 24 % in Viwandani. Slightly more than a fifth (21.5 %) of respondents had eaten one meal or less the previous day (Table 1).

**TABLE 1:** Food security situation by slum of residence

Characteristic	Total	Viwandani	Korogocho	*p* value
Food security				
Secure	14.9	23.9	7.7	<0.001
Mild–moderately insecure	34.9	42.7	28.7	
Severely insecure	50.1	33.4	63.7	
Number of meals^a^				
0–1 meals	21.5	20.7	22.2	<0.001
2 meals	37.4	31.7	42.0	
≥3 meals	41.1	47.6	35.9	
Food variety				
<4 food groups	34.6	31.2	37.2	<0.001
≥4 food groups	65.5	68.8	62.8	

Values are column percentages for characteristics

^a^Number of meals eaten by respondent the previous day

About all households purchased food, with a negligible proportion producing their own food. Most households predominantly purchased raw foods from the market (87 %), while the rest of the households predominantly purchased cooked food from the streets. The level of income was very low, with 71 % earning less than 10,000 Kenya shillings (approx. US$120) per month. Only slightly more than a fifth (22 %) of breadwinners were on formal labor force, with close to half (46 %), being on casual employment, and a substantial proportion (close to a fifth) relying mainly on remittances. Use of suboptimal coping strategies (e.g. skipping meals, trading sex for food or money, etc.), was prevalent in the surveyed households (77 %). There was a high level of either perceived or experienced personal insecurity by household members, which may have an impact on food security with regard to access to the food market or livelihoods. About half of the respondents perceived the status of personal security to be either bad or very bad, while only 14 % perceiving it to be good or very good. Close to 10 % of households had experienced a security shock (either mugging or burglary). Across these measures/factors, the situation was worse in Korogocho compared to Viwandani (Table 2).

**TABLE 2:** Vulnerability related characteristics by slum of residence

Characteristic	Total	Viwandani	Korogocho	*p* value
Main food source				
Purchased raw food from market	86.8	88.9	85.1	0.002
Purchased cooked street foods	13.2	11.1	14.9	
Main source of income				
Formal labor	22.3	32.6	13.9	<0.001
Casual labor	46.2	41.8	49.8	
Self-employed	14.0	11.7	15.9	
Remittance	17.5	13.9	20.5	
Household income				
<KShs 10,000	71.0	61.7	78.6	<0.001
≥KShs 10,000	29.0	38.3	21.4	
Dependency ratio	1.63	1.21	1.98	<0.001
Use of negative coping strategies				
No	23.4	34.8	14.1	<0.001
Yes	76.6	65.2	85.9	
Perceived security status				
Bad	49.5	42.2	55.4	<0.001
Satisfactory	36.1	39.1	33.6	
Good	14.4	18.7	11.0	
Experienced insecurity shock^a^				
No	91.2	93.3	89.5	>0.001
Yes	8.8	6.7	10.5	

Values are column percentages for characteristics

^a^Mugging or burglary

### Factors Associated with Food Security

Factors that were significantly associated with food security after controlling for other factors included level of income, source of livelihood, household size, dependence ratio; illness, perceived insecurity and slum ofs residence. Compared to households with a reported income of less than KES 5000 (∼US$60) per month, the odds of being food secure were two times higher in households with an income of between KES 5,000 and 10,000 and four times higher in households with an income of more than KES 10,000 (*p* < 0.001, respectively). Compared to households whose main source of income was from formal labor, the odds of food security in households whose main source of income was from casual labor, were about 50 % lower (*p* < 0.001), while those households whose main source of income was from own business were about 30 % lower (*p* < 0.1). The households with missing information on income and source of livelihood seem to represent a special group of people since they were more likely to be food secure. Larger households were more likely to be food secure after controlling for other factors, with a 12.5 % increase in the odds of food security per additional member of household (*p* < 0.05). However, households with higher dependency ratio (number of non-working members vs. working members) were less likely to be food secure with about 30 % decrease in food security per one unit increase in dependency ratio (*p* < 0.001). Households with at least an ill household member had approximately 30 % lower odds of being food secure compared to households with no sick household member (*p* < 0.01). Compared to households for whom the respondent perceived that the status of security in the neighborhood to be bad, households whose respondent perceived the security status to be satisfactory had about 1.5 times higher odds of being food secure (*p* < 0.01), while those whose respondent perceived the security status to be good had about three times higher odds of being food secure (*p* < 0.001). Households in Korogocho had about 60 % lower odds of being food secure compared to households in Viwandani (Table 3).

**TABLE 3:** Logistic regression for predictors of food security

Variables	Un-adjusted OR; [95 % CI]	Adjusted OR [95 % CI]
Household income (ref: <KES 5,000 [∼US$60])		
5,000–10,000 [∼US$60–120,000]	2.50*** [1.80; 3.67]	2.00*** [1.37; 2.93]
Above KES 10,000 [above US$120]	5.98*** [4.21; 8.52]	3.99*** [2.73; 5.82]
Missing	3.87*** [2.60; 5.78]	3.10*** [2.02; 4.76]
Source of livelihoods (ref: formal)		
Casual	0.32*** [0.25; 0.40]	0.52*** [0.40; 0.67]
Business	0.39*** [0.28; 0.54]	0.71* [0.49; 1.02]
Scavenging and remittance	0.51*** [0.39; 0.68]	0.95 [0.69; 1.32]
Missing	0.49* [0.22; 1.11]	1.48 [0.58; 3.78]
Household size (continuous)	0.85*** [0.80; 0.89]	1.13** [1.01; 1.25]
Dependency Ratio (continuous)	0.75*** [0.71; 0.81]	0.72*** [0.63; 0.82]
Illness in the household	0.50*** [0.40; 0.62]	0.69*** [0.54; 0.87]
Experienced any shock	0.51*** [0.33; 0.79]	0.76 [1.36; 2.13]
Rate of community security (ref: bad)		
Satisfactory	1.70*** [1.35; 2.14]	1.46 ***[1.14; 1.86]
Good	3.77*** [2.90; 4.88]	2.90*** [2.18; 3.84]
Korogocho residence (ref: Viwandani)	0.26*** [0.21; 0.33]	0.40*** [0.32; 0.51]

*p<0.1, **p<0.05, ***p<0.01

### Experiences with Food Insecurity and Coping Strategies

From the qualitative study, we were able to establish how the community members perceived and experienced food insecurity generally and during crisis times, and the coping strategies they employed. Generally, the qualitative findings on their experiences support results from the quantitative data.

### Food Security During Normal Situation in the Slums

The respondents did not draw a very strong distinction between crisis and non-crisis times but tended to move fluidly between generally and specifically regarding their definition of normal situation and crisis times. This may indicate perhaps that they are dealing with a chronic crisis state, unlike rural populations that tend to have periods of plenty and periods of hardship, slum dwellers have a more daily challenge of how to put food on the table.

#### Food Availability, Affordability and Access

Respondents indicated that food for sale, whether cooked or raw, is readily available in the slum communities. Given the cash-based economy in the slums, this food is usually sold in small portions to enhance affordability. Food was reported to be cheap during normal periods especially in Korogocho, sometimes cheaper than other non-slum settings.

If you pass by this road, you cannot lack a food kiosk; there are so many food kiosks and a chapatti (some type of shallow fried bread) costs five shillings (∼USD 0.06). Food is sold cheaply….On the side of food we are okay. Let alone even going to the food kiosk; if you cook for yourself, for *sukuma wiki* (kale) worth five shillings, you can feed three people. Everything is cheap. (FGD, Young Men, Korogocho)

However, despite food being considered to be relatively cheap in the slum communities, people have limited livelihoods, and are sometimes not able to buy the cheap food. Some respondents indicated that people in the slums just eat enough for bare survival; “we don’t eat quality food but just to fill the empty stomachs”. Some respondents considered one meal per day enough and the norm for adults, sometimes with an additional tea in the morning. For children, in several occasions they were also said to survive on one meal but sometimes the caretakers made all efforts to give them a second meal, such as chips (fried potatoes) worth five Kenya shillings (US$0.06) or a banana. Respondents indicated they struggle to ensure children eat something (even when they themselves go hungry), so that the children do not embarrass them to their neighbors:

Here people don’t have breakfast or lunch…just supper… When we were young, if mum cooked lunch we would have to tell the other children due to the joy we would have. (They laugh) We had gotten used to it and if we cooked lunch, we had to brag about it … Most of the people here only eat supper. Like now look at the time and my child has not had anything. He has not even had tea… He will only eat supper and he is used to it. You just buy him a banana at five shillings and that’s it; and he will not cry because he is used to it. (FGD, Unmarried young women, Viwandani)

#### Food Variety and Quality

Food variety and quality was reported as a major challenge and respondents often indicated relying on one type of food. People were said to rely a lot on maize meal or *ugali* as it is locally called, usually eaten with *sukuma wiki* (kale). Foods like meat were said to be a luxury item and unaffordable. Some respondents also indicated that some foods sold in the slums are of lower quality compared to foods sold in non-slum settings. For example, meat sold in the slums was said to be of lower quality than that sold in the non-slum estates, despite the fact that it costs the same; “there is high class and low class meat; low class goes to the “ghetto” while high class goes to the estates…and the price is the same”.

Most people don’t really care what kind of food they are eating because they rely on what they can afford. You can find that in a household they have been eating *ugali (maize meal)* and *sukuma wiki* (kale) for 1 week continuously and it is not because they want to, but they are not able to afford to change the diet.

…And I can tell you there are many people here who sleep on porridge only. You find that they drank porridge in the morning, never had anything at lunch time and then in the evening they make the same porridge. (FGD, Older Men, Korogocho)

#### Use of Street Foods

Consuming foods cooked on the streets was said to be a common occurrence, with seemingly little choice for otherwise. Cooking food was considered expensive particularly using charcoal or firewood. People were said to mainly use paraffin as cooking fuel, meaning that increasing fuel prices has direct effects on food security. Given their financial limitations, people were said to cook small portions of food…for just one meal, hence the diseconomies of scale. Buying street food sold on the roadside was considered cheaper than cooking, as when one cooks, they have to buy the raw food and fuel, which ends up being expensive. This is despite the fact that they consider street foods to be of lower quality in terms of hygiene in preparation and the nutrients in the food:

… For example, these women who cook on the roadside, they don’t clean the food before cooking at all… I think they also use bicarbonate of soda so that it can cook fast and when you eat such kind of food, it affects the knees and generally makes the bones to be weak… In fact when you walk you hear them knocking each other knock, knock, knock. (Young Men, Viwandani)

You find in the kiosks for example they cook with dirty water. They fetch water from the sewage and come and use it for cooking and you know people just eat there because they feel it is cheap. (FGD, Unmarried young women, Viwandani)

School feeding programs was a notable source of food for children, and it was reported that some parents wait for children to carry food home from school.

### Food Security During Crisis Times in the Slums

Though slum dwellers live in chronic poverty with high level of vulnerability to food insecurity even during normal times, they experience high level of vulnerability to food insecurity during crises times. The nature of crisis determines the level and nature of vulnerability to food insecurity. For example during some crises, availability and physical access to food in the market is highly affected, while in some, financial accessibility would be the main problem. For example, droughts result in limited availability of foods in the market, hence high prices, while insecurity-related crises result in limited availability, accessibility and affordability of food. When asked to recall a recent crisis and their experiences, respondents gave narratives of their experiences with food security during the 2007/2008 postelection violence in Kenya that followed the disputed presidential elections that ran for a couple of months.

The 2007/2008 postelection violence was reported as having highly impacted the availability of food in the market, physical access to that food and affordability of food due to limited job opportunities, which impacted on income flow for the household. This was particularly exacerbated by rise in the level of personal insecurity. The violence and insecurity meant that the residents, who are mainly casual laborers, could not access jobs, so their purchasing power was highly reduced. Traders could not bring food into the slums for sale or they could not open their food stalls at all. This was due to problems in accessing food for sale from rural areas, fear of opening food stores/stalls due to looting and arson, and limited clients’ access to the market due to rampant insecurity levels in the slum communities. Common commodities like maize (and consequently maize flour), the main staple food reportedly disappeared from the local market during the crisis. Fresh vegetables were mostly non-existent. The scarcity of food in the market escalated the price of food that was available to unaffordable levels, often double the normal prices or higher. Vulnerability to food insecurity was exacerbated by the fact that slum dwellers mainly survive on daily wages, thus could only buy food enough for just a day, and had no stock of food in their homes when the crises erupted. These residents therefore experienced intense hunger as narrated below:

There were no jobs, there was no transport, some other roads had been closed and thus there was no food. We experienced severe hunger because maybe you have money in your pocket but you could not buy food. We suffered a lot and had it continued, we would have really suffered. … What I saw most was hunger which affected a lot of people especially because there was no job and even if you find a place where there are some vegetables, you cannot afford. (FGD, Older men, Viwandani).

Because I could not go to work neither could the rest, so all of us were hungry…People had already finished the stock they had in their houses…You could go out of the house and the porridge you had in the house was finished…There was too much hunger such that people would attack a cow; there were some men who used to rear cows here, they would attack a cow and cut it into pieces alive (even without slaughtering it). Even the pigs; there was no chance of slaughtering; just cutting them and running away with the meat…That was hunger… There were high levels of hunger because even if you had money, you could not find a place to buy food. If you had money in the bank, the banks were closed. (FGD, Older Men, Korogocho)

Young children and their mothers bore the brunt of the crisis as it was survival for the fittest even with food aid. Mothers did not have enough food to produce adequate breast milk, and neither were there available breast milk substitutes and supplemental foodstuff to feed the children. Lack of food during the crisis had have adverse effects especially on the children including severe malnutrition and death. The following narrative portrays the vulnerability of women and children during times of crises:

It was bad because we could not get milk…in fact a lot of children died. You find that a woman is breastfeeding but cannot get milk and you know if you don’t drink milk, you cannot be able to produce enough breast milk…When the relief (food aid) items come, you queue to go and get some and then the youths come and take everything away. While you are waiting in the queue, they have already gone in and taken everything for themselves. Now children had a lot of problems…they grew thin. They did not get enough food…If it is milk, there wasn’t any. You could be sick and were advised not to breastfeed the child; that child would get problems because one could not even get milk to buy for the child…Another thing is that the hospitals had been closed and if a child was sick, he could just die in the house because all the hospitals were closed. There was no place you could take the child to get treatment. (FGD, Female Heads of Households, Korogocho)

### Coping Strategies

According to the respondents, coping strategies employed as far as food insecurity is concerned are similar during crisis and during non-crisis times, only used more often during a crisis. The most commonly used set of coping mechanisms was reducing food intake, in terms of quantity, quality, diversity and frequency.^25^ Buying precooked food on the streets, was also said to be a very common strategy for poor community members as this allowed them to save on cooking fuel. Buying poorer quality food, particularly old/expired vegetables and fruits was also a common strategy as these were cheaper than fresh items. Others were said to rely on scavenging for thrown away and expired foods from the local market or dumpsites, either buying it from vendors or they scavenge for it themselves. Prioritizing children over adults was also said to be a commonly used coping strategy. Borrowing of food from neighbors or friends was also reported as a normal practice.

…and we have this other one called *Anyona*…It is some bread that comes from factories; broken bread (bread pieces/crumbs)…Most of the people instead of buying the full bread from the shop, people go and buy the *Anyona* in a 2Kg pack…Which is sold cheaply, so that they can come and eat it as bread. The broken bread from the factory is sold at Regio Maria down there. So people walk from all the way here and they go and get the *Anyona* and use it as bread; so that they may tell the children that they have eaten bread at home. (KII, Church Leader, Korogocho)

## Discussion

This study has clearly portrayed the experiences of food insecurity in urban poor settings, illustrating the vulnerability to food insecurity that the urban poor communities experience in their daily lives and during times of crises. The study shows that during “normal times”, food insecurity in the slums is very high with 85 % of households experiencing mild to severe food insecurity. The situation could only be worse during periods of acute crises. The narratives clearly demonstrate the high level of vulnerability to food insecurity among the urban poor during times of crises. As in other studies in the same setting, our multivariate analyses identified factors associated with food security in the slum setting including level of income, source of livelihood, household size, dependence ratio; illness, perceived insecurity and location. Given the cash-based economy in the slums, and the fact that food is purchased on a day-to-day and sometimes meal-to-meal basis in this population, compared to many of the other major expenses such as rent, school fees and business inputs, food expenditure is highly malleable and can easily be decreased to cover shortfalls. Suboptimal coping strategies are employed to cope with food insecurity and to a higher degree during times of crises. These include reducing food intake in terms of quality and quantity, prioritizing children over adults, buying street foods and scavenging for foods from markets or dumpsites. A previous study indicates that there are limited social support networks for people who suffer food insecurity in the slums.^25^

Faye and colleagues reported similar findings in their 2011 paper using data collected between 2006 and 2008 through the Nairobi Urban Health and Demographic Surveillance System in the two study slums.^26^ However, they used one- and two-parameter item response theory (IRT) models to classify food insecurity into five categories: (i) food-secure; (ii) food-insecure without hunger; (iii) food-insecure with adult hunger; (iv) food-insecure with child hunger; and (v) food-insecure with both adult and child hunger as opposed to the four categories in our study using the HFIAS criteria. They found that only a fifth of urban poor households were food secure, and 50 % of households were classified as food insecure with child and adult hunger, which may be equivalent to the 50 % severe food security in our study. In line with our study, they also found a disparity in the levels of food insecurity between the two slums, with Korogocho being worse off. In their paper, they indicated a rising trend in the levels of food insecurity over time between 2006 and 2008. The current study has gone a step further to obtain qualitative narratives from the slum residents in order to clearly understand experiences of food insecurity in these urban poor settings during normal and crises situations.

Economic crises that may be as a result of various causes including political instability, environmental conditions among others have been implicated in rise in food insecurity in various settings.^27^^–^ 29 The rise in global food prices since 2007 signals a food insecurity crisis among the world’s poor, and the high levels of food insecurity reported in this paper may be a manifestation of this phenomenon, among other frequent crises that affect urban poor residents, including crises due to political instability.^28^^,^ 29 In line with our study, high levels of food insecurity have also been documented in other urban poor settings in the developing world.^30^^,^ 31 For example, Gopichandran and colleagues in 2010 found that only 25 % of households in a densely populated urban area in Southern India were food secure.^31^

Results from our study indicate a major deviation from the declaration of the World Food Summit of 1996 that food security exists “when all people at all times have access to sufficient, safe, nutritious food to maintain a healthy and active life”, and that everyone has a right to food security.^32^ Most of the households in the urban slums surveyed were food insecure, while half of the households were actually severely food insecure. The narratives paint a very grave picture of food insecurity in these settings. Dietary diversity seems to be rare and people are often forced to repeat the same meal over and over again. The situation was reported as severe during crises times, when not only are people unable to afford and access safe and nutritious food, they are forced to acquire any food through socially unacceptable means for bare survival such as “cutting live animals and running away with the meat”. The narratives indicate that slum dwellers are not only living in chronic poverty, but also live on the edge, and any disruption of the system can put them under extreme vulnerability to food insecurity.

Findings from this study indicate the need for measures to address the food security situation in urban poor settings and to the particular need to mitigate effects of crises on food security in these settings. To address the chronic poverty situation in urban areas, there is need to establish sustainable livelihood opportunities for the urban poor. With regards to agricultural productivity, urban residents depend almost entirely on rural areas for their food. Urban agriculture may be a promising strategy to address household food security as it has been seen to improve food diversity and livelihoods, but on a small scale given lack of adequate farming land in urban areas.^33^^–^ 35 This brings to the fore the challenge of access to land for landless urban poor in Kenya. Beyond land however, there is need to take care of potential health risks associated with urban agriculture particularly chemical waste due to use of waste water.^36^ Given that food security is a human right,^32^ there is need for consideration of social protection measures including cash transfers and food aid by the government and humanitarian agencies for the urban poor, particularly for the most vulnerable households and during crises times. Finally, setting up early warning systems to detect impeding food security crisis is also important for emergency preparedness in urban poor settings, as these are rare in urban areas.^37^ Further research is also needed to identify the most effective ways of addressing urban food insecurity.

In summary, this study describes the deeply intertwined nature of chronic poverty and acute crisis, and the consequence on household food insecurity in urban slum settings. The limited income and livelihood opportunities result in households being extremely vulnerable to food insecurity, particularly during periods of crisis, and resorting to consequently employing negative coping strategies. Effective response to address vulnerability to household food insecurity among the urban poor should focus on both the underlying vulnerabilities of households due to chronic poverty and the impacts of acute crises on these households. Measures may need to be put in place by the government and humanitarian agencies to detect impeding crises for emergency preparedness and to cushion slum dwellers against predictable adverse impacts of crises on food security.

## Appendix

### Questions Contributing to the Household Food Insecurity Access Scale (HFIAS)

For each question, the response categories were as below:

- 0 = Never
- 1 = Rarely
- 2 = Sometimes
- 3 = Often

[CIRCLE THE APPROPRIATE RESPONSE]

1. In the past 4 weeks, did you worry that your household would NOT have enough food? How often?
2. In the past 4 weeks, were you or any household member NOT able to eat the kinds of food you preferred because of a lack of resources? How often?
3. In the past 4 weeks, were you or any household member have to eat a limited variety of foods due to lack of resources? How often?
4. In the past 4 weeks, did you or any household member have to eat a smaller meal than you felt you needed because there was NOT enough food?
5. In the past 4 weeks, did you or any household member have to eat fewer numbers of meals in a day because there was NOT enough food?
6. In the past 4 weeks, was there ever NO food of any kind to eat in your household because of lack of resources to get food? How Often?
7. In the past 4 weeks, did you or any household member go to sleep at night hungry because there was NOT enough food? How often?
8. In the past 4 weeks, did you or any household member go a whole day and night without eating anything because there was NOT enough food?

## References

[1] World Bank. *African development indicators*. Washington DC: The World Bank, 2012/2013.

[2] UNHABITAT (2008). State of the world’s cities 2010/2011: bridging the urban divide.

[3] African Population and Health Research Center (2002). Population and health dynamics in Nairobi informal settlements.

[4] African Population and Health Research Center (2002b). Health and livelihood needs of residents of informal settlements on Nairobi City. Occasional study report 1.

[5] Magadi M (2004). Maternal and child health among the urban poor in Nairobi. Kenya Afr Popul Stud.

[6] Kimani-Murage EW, Ngindu AM (2007). Quality of water the slum dwellers use: the case of a Kenyan slum. J Urban Health.

[7] Ahmed AU, Hill RV, Smith LC, Wiesmann DM, Frankenberger T. The world’s most deprived: characteristics and causes of extreme poverty and hunger. *Intl Food Policy Res Inst*. 2007.

[8] Amendah D, Buigut S, Mohamed S. Coping strategies among urban poor: evidence from Nairobi, Kenya. *PLoS One* In press. 10.1371/journal.pone.0083428 PMC3888389 24427272

[9] Garrett J. Living life: overlooked aspects of urban employment. *FCND Discussion paper 171*; 2004. URL: http://www.ifpri.org/sites/default/files/publications/fcndp171.pdf. Accessed August 2014.

[10] Beall J, Fox S. Urban poverty and development in the 21st century: towards an inclusive and sustainable world: Oxfam GB, Development Studies Institute, London School of Economics 2006.

[11] Cohen MJ, Garrett JL (2010). The food price crisis and urban food (in)security. Environ Urban.

[12] Haddad L, Ruel MT, Garrett JL (1999). Are urban poverty and undernutrition growing? Some newly assembled evidence. World Dev.

[13] Galal O, Corroon M, Tirado C (2010). Urban environment and health: food security. Asia Pac J Public Health.

[14] Armar-Klemesu M. Urban agriculture and food security, nutrition and health. Growing cities, growing food urban agriculture on the policy agenda. 2000; 99–118.

[15] Maxwell D (1999). The political economy of urban food security in Sub-Saharan Africa. World Dev.

[16] Ruel MT, Garrett JL, Hawkes C, Cohen MJ (2010). The food, fuel, and financial crises affect the urban and rural poor disproportionately: a review of the evidence. J Nutr.

[17] Martin-Prevel Y, Becquey E, Tapsoba S (2012). The 2008 food price crisis negatively affected household food security and dietary diversity in urban Burkina Faso. J Nutr.

[18] Ruel M, Garrett J, Morris S, Maxwell D, Oshaug A, Engle P, Slack A, Haddad L. Urban challenges to food and nutrition security: a review of food security, health and caregiving in the cities. *FCND Discussion paper no. 51*. 2008.

[19] Mutua MK, Kimani-Murage E, Ettarh RR. Childhood vaccination in informal urban settlements in Nairobi, Kenya: who gets vaccinated? *BMC Public Health*. 11(1):6. 10.1186/1471-2458-11-6 PMC3024932 21205306

[20] Emina J, Beguy D, Zulu EM (2011). Monitoring of health and demographic outcomes in poor urban settlements: evidence from the Nairobi Urban Health and Demographic Surveillance System. J Urban Health.

[21] Schofield L, Mohamed SF, Kimani-Murage EW, et al. Spotting the invisible crisis: early warning indicators in urban slums of Nairobi Kenya. Special focus on urban food security & nutrition; *Field exchange*. 46: Special focus on urban food security & nutrition. p55. 2013. URL: http://www.ennonline.net/fex/46/spotting. Accessed August 2013.

[22] Coates J, Anne S, Paula B (2006). Household Food Insecurity Access Scale (HFIAS) for measurement of household food access: indicator guide.

[23] Strauss AL, Corbin JM (1990). Basics of qualitative research: grounded theory procedures and techniques Newbury Park.

[24] Ryan GW, Bernard HR (2003). Techniques to identify themes. Field Methods.

[25] Amuyunzu-Nyamongo M, Ezeh AC (2005). A qualitative assessment of support mechanisms in informal settlements of Nairobi, Kenya. J Poverty.

[26] Faye O, Baschieri A, Falkingham J, Muindi K. Hunger and food insecurity in Nairobi’s slums: an assessment using IRT models. *J Urban Health*. 88(Suppl 2): 235–55. 10.1007/s11524-010-9521-x PMC3132228 21234694

[27] Soekirman A (2001). Food and nutrition security and the economic crisis in Indonesia. Asia Pac J Clin Nutr.

[28] Hadley C, Linzer DA, Belachew T, Mariam AG, Tessema F, Lindstrom D (2011). Household capacities, vulnerabilities and food insecurity: shifts in food insecurity in urban and rural Ethiopia during the 2008 food crisis. Soc Sci Med.

[29] Hadley C, Stevenson EG, Tadesse Y, Belachew T (2012). Rapidly rising food prices and the experience of food insecurity in urban Ethiopia: impacts on health and well-being. Soc Sci Med.

[30] Agarwal S, Sethi V, Gupta P, Jha M, Agnihotri A, Nord M (2009). Experiential household food insecurity in an urban underserved slum of North India. Food Sec.

[31] Gopichandran V, Claudius P, Baby LS, Felinda A, Mohan VR (2010). Household food security in urban Tamil Nadu: a survey in Vellore. Natl Med J India.

[32] World Food Summit. *Rome declaration on world food security*. 1996. URL: http://www.fao.org/docrep/003/w3613e/w3613e00.HTM. Accessed August 2014.

[33] Maxwell DG (1995). Alternative food security strategy: a household analysis of urban agriculture in Kampala. World Dev.

[34] Yeudall F, Sebastian R, Cole DC, Ibrahim S, Lubowa A, Kikafunda J (2007). Food and nutritional security of children of urban farmers in Kampala, Uganda. Food Nutr Bull.

[35] Wills J, Chinemana F, Rudolph M (2010). Growing or connecting? An urban food garden in Johannesburg. Health Promot Int.

[36] Gallaher CM, Mwaniki D, Njenga M, Karanja NK, WinklerPrins AM (2013). Real or perceived: the environmental health risks of urban sack gardening in Kibera slums of Nairobi, Kenya. EcoHealth.

[37] Emergency Nutrition Network. Special focus on urban food security & nutrition. *Field exchange*. 2013. URL: http://files.ennonline.net/attachments/1613/fx-46-web.pdf. Accessed August 2014.

